# Investigation of periodic characteristics of perturbed flow over a slender body

**DOI:** 10.1016/j.heliyon.2023.e16194

**Published:** 2023-05-13

**Authors:** Li Zhao, Yankui Wang, Zhongyang Qi

**Affiliations:** Ministry-of-Education Key Laboratory of Fluid Mechanics & Aircraft and Propulsion Laboratory, Ningbo Institute of Technology, Beihang University, Beijing 100191, China

**Keywords:** Slender body, Bluntness, Asymmetric flow, Separation, Periodic characteristics

## Abstract

The asymmetric flow over a slender body was particularly sensitive to the nose at a high angle of attack (AoA). Two patterns of separation occurred on the noses of the pointed-nosed slender body and blunt-nosed slender body as open- and close-type separation, respectively. The effects of the bluntness were investigated at high AoA (*α* = 50°) to clarify the evolution of the separated pattern from open-to close-type separation by the nose and by the periodic characteristics of perturbed flow. Wind tunnel experimental tests were conducted to investigate the periodic characteristics of asymmetric flow at a Reynolds number *Re*_*D*_ = 1.54 × 10^5^, based on incoming free-stream velocity (*U*_*∞*_) and the diameter (*D*) of the model. A particle was attached to the tip of the nose to induce the perturbed flow and attain a definite and predictable asymmetric flow in experimental tests. The pressure scanning and surface oil-flow visualization techniques were used to capture the pressure distributions and flow separations. The major findings were that axial flow increases with the increase of bluntness, resulting in open-type separation turning into close-type separation, and the perturbation moved from downstream to upstream of starting points of the separation line. The critical bluntness of separation pattern switching from open-type to close-type located between 1.5 and 3. Thus, the management of perturbation on asymmetric flow pattern switched from directly participating in separation to influencing separation through micro-flow. Therefore, the locations of perturbation and starting points of the separation line were closely related to asymmetric flow management by perturbation, then affecting the periodic characteristics of perturbed flow.

## Nomenclature

*α*: angle of attack
*D*: diameter of after body
*B*: bluntness
*R*: radius of nose
*d*: diameter of perturbation
*x*: distance from origin (0, 0)
*L*_*p*_: distance between perturbation and nose apex
*C*_*y*_: sectional side-force coefficient
*F*_*y*_: sectional side-force
*ρ*_*∞*_: mass density of air
*C*_*p*_: pressure coefficient
*A*: cross section area of the body
*γ*: meridian angle
*θ*: circumferential angle
*θ*_*s*_: sectional angle
*U*_*∞*_: freestream velocity
*Re*_*D*_: Reynolds number based on *D* and *U*_*∞*_
*θ*_*separation*_: separation location

## 1 Introduction

The asymmetric flow separation occurred over a slender body at a high angle of attack (AoA) [1,2]. In addition, the slender body was the basic geometry of fighter aircraft and missiles, hence, the highly random asymmetric flow was coupled with wings and induces a yawing moment [3], which was even largely great beyond the control by the rudder of a missile [2,4,5]. In addition, the development and bursting of asymmetric vortices were sensitive to the structure of the wings and the coupling position at high AoA [6,[7], [8]]. During side-force and yawing, the controllability of the aircraft and missile was reduced, thereby resulting in a failed mission because the desired tracks of the fighter aircraft and missiles were changed. Hence, it was significant to investigate the asymmetrical flow over a slender body at high AoA.

The asymmetric flows phenomenon has been extensively studied to reveal the flow mechanism. Pick [9] first proposed that the asymmetry of boundary separating caused the asymmetric vortex flow-field. In other words, the micro asymmetries and geometrical imperfections at the nose of the slender body induced the asymmetric flow. Considering that the machining tolerance of the model's nose was inevitable, Levy et al. [10] applied a micro perturbation onto the tip of the slender body by numerical simulation. They found that the asymmetric flow at high AoA was induced by the artificial micro perturbation, but turned into symmetric when the artificial micro perturbation was canceled. The follow-up experimental studies [[11], [12], [13]] provided experimental evidence. To sum up, the asymmetric flow over a slender body at a high AoA was especially sensitive to the model's nose [14].

Considering the sensibility of the asymmetric flow to the nose, considerable studies have been devoted to revealing the relationship between the parameters of the nose and the asymmetric flow pattern. For reducing the asymmetry of the asymmetric flow, Ericsson [11] attached a nose boom onto the tip of a slender body and he found that the asymmetric flow occurred at a higher AoA and the maximum side force decreased by 60%. Meanwhile, Rao [15] pasted a pair of transitional wires onto both sides of the nose of the slender body to disrupt the flow separation, thereby resulting in no obvious vortex structure occurring, hence, the side force was reduced. Chen [16] and Deng et al. [[17], [18], [19]] employed a 0.2 mm diameter particle onto the tip of the slender body to disturb the asymmetric flow at high AoA. The asymmetric flow pattern was associated with the parameters of the particle. Meng [20] embedded a plasma actuator on the nose of the slender body to generate a micro-stream on the nose and subsequently influenced the asymmetric flow. A synthetic jet at the nose was introduced by Wang [21] for affecting the asymmetric flow. In addition, there was more research on the influence of nose parameters on the asymmetric flow, including nose blunting [[22], [23], [24], [25]], blowing/suction acting on the nose [26,27], nose deflection [28], and perturbation issued at the nose [29,30]. These number of research have obtained limited achievements and still require to be verified in a broad range of flow parameters.

**Fig. 1:**
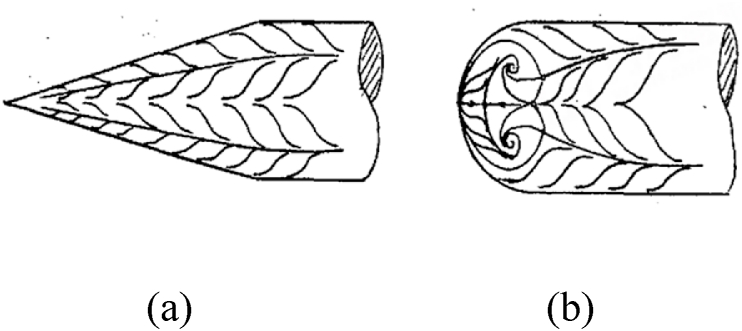
Schematic diagrams of separations over the slender bodies with different nose shapes. (a) open-type separation over a pointed-nosed slender body; (b) close-type separation over a blunt-nosed slender body [33].

The above studies stated that nose blunting was the simplest and most viable technology, and it can be further extended to affect the bluntness of the asymmetric flow over a slender body at high AoA. Nose blunting delayed the onset of flow asymmetry [[22], [23], [24]]; however, the benefits were only demonstrated within a restricted range of flow parameters [31]. Wang [24] found another fundamental phenomenon for nose blunts. In addition, as shown in Fig. 1, two different types of stable separation lines were presented on slender body of the pointed- and the blunt-nose, these were open- (Fig. 1(a)) and close-type separation (Fig. 1(b)) for slender body of the pointed- and blunt-nose, respectively. Moreover, for the pointed-nosed slender body characterized by open-type separation, the asymmetric flow, and its corresponding side-force changed in a double period when the roll angle increased from 0° to 360° [17]; however, the single period was presented for the blunt-nosed slender body characterized by close-type separation [32].

It was evident from the aforementioned review of previous work that there were three unresolved issues regarding the suppression flow asymmetry over a slender body by nose blunting. The first was the weakening mechanism of nose blunting on flow asymmetry. The second issue was the mechanism of switching from open-to close-type of separation with nose blunting. The third issue was the reason why the perturbed flows over pointed- and blunt-nosed slender body have the double and single periodic characteristics respectively. In order to address these three issues, the focus of this work was on the influence of the bluntness on the separation flow over a slender body at a high AoA (*α* = 50°). Wind tunnel tests were carried out with a Reynolds number *Re*_*D*_ = 1.54 × 10^5^. The separation lines, flow structures, as well as pressure distribution were analyzed at length. The test model and approaches were introduced in Section 2, the results were displayed and discussed in Section 3, and this work was concluded in Section 4.

## 2 Test model and approaches

### 2.1 Test model

**Fig. 2:**
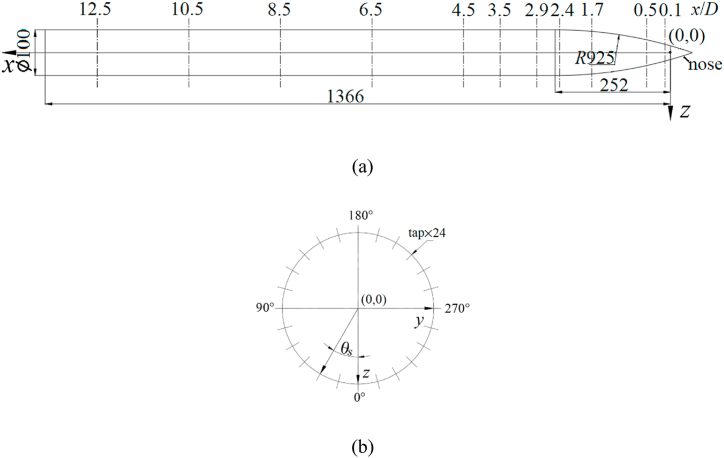
Schematic diagram showing the test model of a slender body, together with symbol designations. (a) side view; (b) end view. Units of length scale are in mm.

**Fig. 3:**
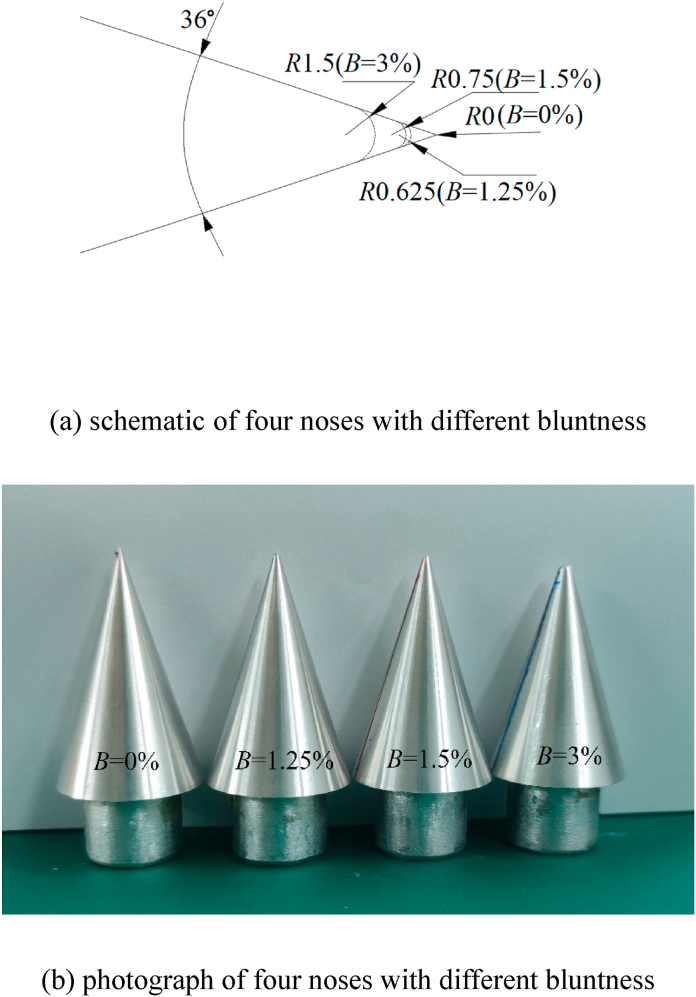
Four noses with different bluntness (*B*) and definitions of their bluntness.

Fig. 2 presented the test model of the slender body. There were three specific parts in the test model that included the circular cone nose, the cylindrical slender afterbody, and the arched part joining the former two (Fig. 2(a)). The cylindrical slender afterbody had a diameter of *D* = 100 mm and the length was 1084 mm (= 10.84 *D*). Meanwhile, the circular cone nose had an apex of 36° and a length of 46 mm (= 0.46 *D*) and it can be replaced by noses with different bluntness (*B*), as shown in Fig. 3. Four noses with different bluntness were used in this study. Fig. 3(a) presents the schematic of four noses with different bluntness and Fig. 3(b) is the photograph of four noses. The bluntness referred to the ratio of the diameter of nose to the diameter of afterbody (*D*). The length of the arched part was 252 mm (= 2.52 *D*) and the arch's radius was 925 mm (= 9.25 *D*). The leading edge of the arched part was the origin of the coordinate system, with *x*-direction along the symmetric axis of the slender body, and *y*- and *z*-directions as the horizontal and vertical axes, respectively, in the plane perpendicular to the symmetric axis. In addition, Fig. 2 presented the test model with pressure taps, which were utilized for the pressure measurements. The model included 11 tapping stations, each with 24 evenly-spaced taps around the cylinder circumference. The diameter of each pressure tap was 1 mm, and its circumferential locus was indicated by the angle *θ*_*s*_ of the negative *z*-axis and the sectional radius corresponding to the pressure location measured (Fig. 2(b)).

**Fig. 4:**
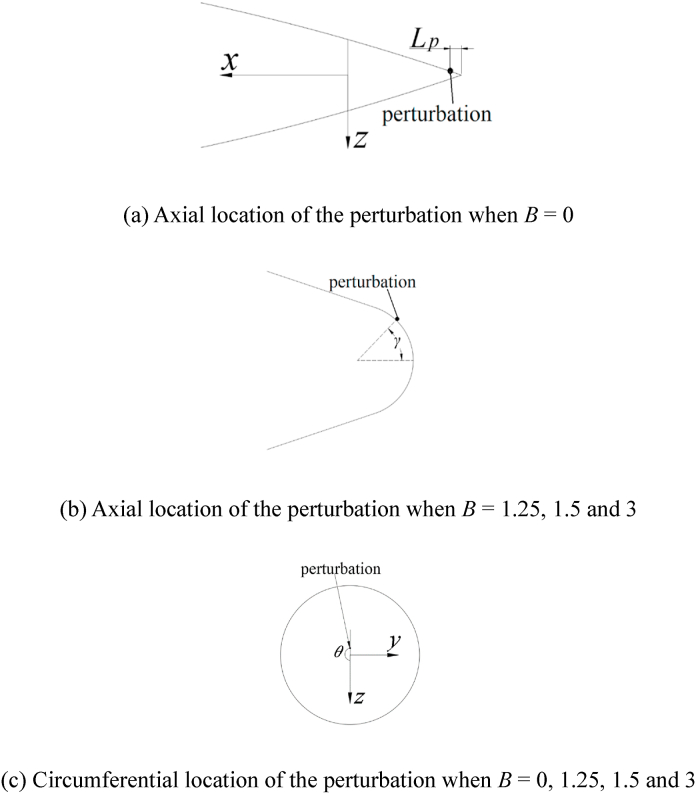
Definition of artificial micro perturbation.

Based on the previous studies [16,17,32]; on the asymmetric flow over a slender body, the artificial perturbation near the tip of the slender body eliminates the uncertainty of flow and provide a stable asymmetric flow structure to study. In the present study, a micro particle with a diameter of *d* = 0.004*D* was taken as the artificial perturbation onto the nose of the slender body. As shown in Fig. 4, for *B* = 0, the axial location of the perturbation was designated by *L*_*p*_ (Fig. 4 [a]), which was the *x*-directional distance from the tip of the pointed nose to the location of the perturbation. Meanwhile, *L*_*p*_ was set to 1.5 mm (= 0.015 *D*). For other *B*, the meridian angle *γ*, that was the angle of the negative *x*-axis to the nose radius corresponding to the perturbation location, was used to define the axial location of the perturbation (Fig. 4 [b]). In addition, *γ* was set as 10°. The circumferential location of the perturbation was denoted by *θ*, which was the angle of the *z*-axis to the sectional radius that corresponded to the perturbation location (Fig. 4 [c]).

### 2.2 Wind tunnel tests

**Fig. 5:**
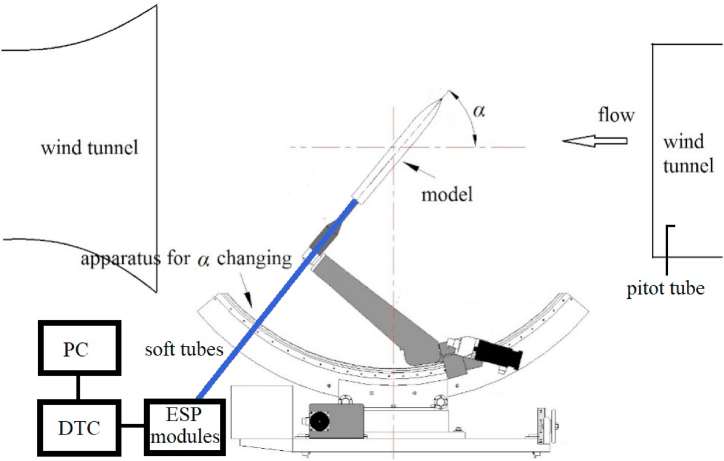
Setup of the test model in a wind tunnel.

Experiments were completed in a low-speed open-circuit wind tunnel, which had a test section with a 2.5-m-long square test section of 1.5 m × 1.5 m. This wind tunnel had a speed range of 2 m/s–60 m/s, with a freestream turbulence intensity of ≤0.08%. Fig. 5 presented the set-up of the test model in the wind tunnel. A pitot tube was placed near contraction exit of the tunnel to monitor the wind speed. The slender body was sting-mounted on a supporting mechanism that allowed the AoA to be adjusted from 0° to 70°, leading to a maximum block of 4%. The AoA, which was the incident angle of the oncoming flow to the *x*-direction, was denoted by *α*. The minimum distance from the leading edge of the slender body to the tunnel contraction exit was 0.15 m. The Reynolds number in all the following tests was *Re*_*D*_ = 1.54 × 10^5^, based on *D* and *U*_*∞*_, with *U*_*∞*_ being the incoming freestream.

A pressure scanning system from the PSI company was used to acquire the pressure distributions around the test model. This system was formed by DTC initium modules and four ESP (electronic scanning pressure) modules (each has 64 channels) with an uncertainty coefficient of *σ*_*i*_ = 0.764%. Each tap was set as *i*，Number of measurement was set as *j*, the mean value:

(1) $$\bar{x_{i}}=\frac{1}{n}\sum_{j=1}^{n}x_{ij}$$

The standard deviation of each tap:

(2) $$\sigma_{i}={\left[\frac{1}{n-1}{\sum_{j=1}^{n}\left(x_{ij}-\bar{x_{i}}\right)}^{2}\right]}^{\frac{1}{2}}$$

The standard deviation of the total：

(3) $$\sigma ={\left[\frac{1}{k}\sum_{i=1}^{k}\sigma_{i}^{2}\right]}^{\frac{1}{2}}$$

(4) $$\sigma_{i}={\left[\sum_{i=1}^{k}{\sum_{j=1}^{n}\left(x_{ij}-\bar{x_{i}}\right)}^{2}/k\left(n-1\right)\right]}^{1/2}$$

Where *k* was the number of taps, *n* was the number of repeated measurements. Each pressure tap on the test model was connected to one channel on the ESP module with a soft tube, which had an inner diameter of 1 mm. The DTC Initium module, of which the maximum sampling frequency was 600 Hz, received the measured data from the ESP modules through the data wires and subsequently sent the data to the computer. The sampling frequency and record time in the pressure measurements were 50 Hz and 12 s in this study, respectively. All curves fitted to the data points in this paper were fit using a spline fitting method. Table 1 summarized the pressure measurement cases in this paper.

**Table 1:** Pressure measurements.

*B* = 0	*B* = 1.25	*B* = 1.5	*B* = 3		
*θ* = 0°–360° (Δ*θ* = 15°)	*θ* = 0°–360° (**Δ***θ* = 15°)	*θ* = 0°–360° (**Δ***θ* = 15°)	*θ* = 0°–360° (**Δ***θ* = 15°)	*θ* = 0°–360° (**Δ***θ* = 15°)	*θ* = 0°–360° (**Δ***θ* = 15°)
***L***_***p***_**= 0.015*D***	*γ* = 10°	*γ* = 10°	*γ* = 10°	*γ* = 75°	*γ* = 75°
***d* = 0.004*D***	*d* = 0.004*D*	*d* = 0.004*D*	*d* = 0.004*D*	*d* = 0.004*D*	*d* = 0.01*D*

Oil-flow visualization was conducted to present the surface flows. This technique was applied to localize the separations or reattachments by identifying the converging or diverging stripes. In this work, TiO2, silicone oil, and aviation kerosene were admixed in the ratio of 1:10:20 to present the separation lines. Photographs of the surface oil flow visualization were taken by using a digital single lens reflex camera (Nikon, D70) with a zoom lens (Nikon, AF-S DX100-300 mm) to capture the final flow pattern. The resolution was set to 6,100,000 pixels. This technique was extremely helpful in determining the regions of separations or reattachments by identifying converging or diverging streaks [34]. For example, Ramakrishna et al. [35] adopted this technique to analyze the effect of bluntness on characteristics of flow over a slender body. Moreover, Merzkirch [36] described this experimental visualization technique in depth.

### 2.3 Aerodynamic force coefficients

The aerodynamic coefficients used were defined as follows:

(1) Sectional side-force coefficients *C*_*y*_

(5) $$C_{y}=F_{y}/\left(0.5\rho_{\infty }U_{\infty }^{2}D\right)$$

where *F*_*y*_ is sectional side-force and is defined as $F_{y}=\int_{0}^{2\pi }\left(0.5p_{i}D\;\sin\;\theta_{s}\right)d\theta_{s}$, and *p*(*i*) is the static pressure measured at the *i*th tap.

(2) Pressure coefficient *C*_*p*_

(6) $$C_{p}=p\left(\theta_{s}\right)/\left(0.5\rho AU_{\infty }^{2}\right)$$

where *p* (*θ*_*s*_) is the static pressure obtained at the location corresponding to *θ*_*s*_ and *A* is the cross-section area of the slender body.

## 3 Results and discussion

This section presented and discussed the periodic characteristics of perturbed flow over the slender body at a high AoA. Based on the previous studies [37,38], a diameter particle of *d* = 0.004 *D* was initially applied onto the nosed tip to perturb the asymmetric flow, thereby resulting in a certain flow to be obtained. The major effects of the bluntness (*B*) on the perturbed flows and its corresponding flow separations were first discussed, followed by the effect mechanics of bluntness on the perturbed flow. They were presented by analyzing the periodic characteristics.

### 3.1 Effects of bluntness on the perturbed flow

**Fig. 6:**
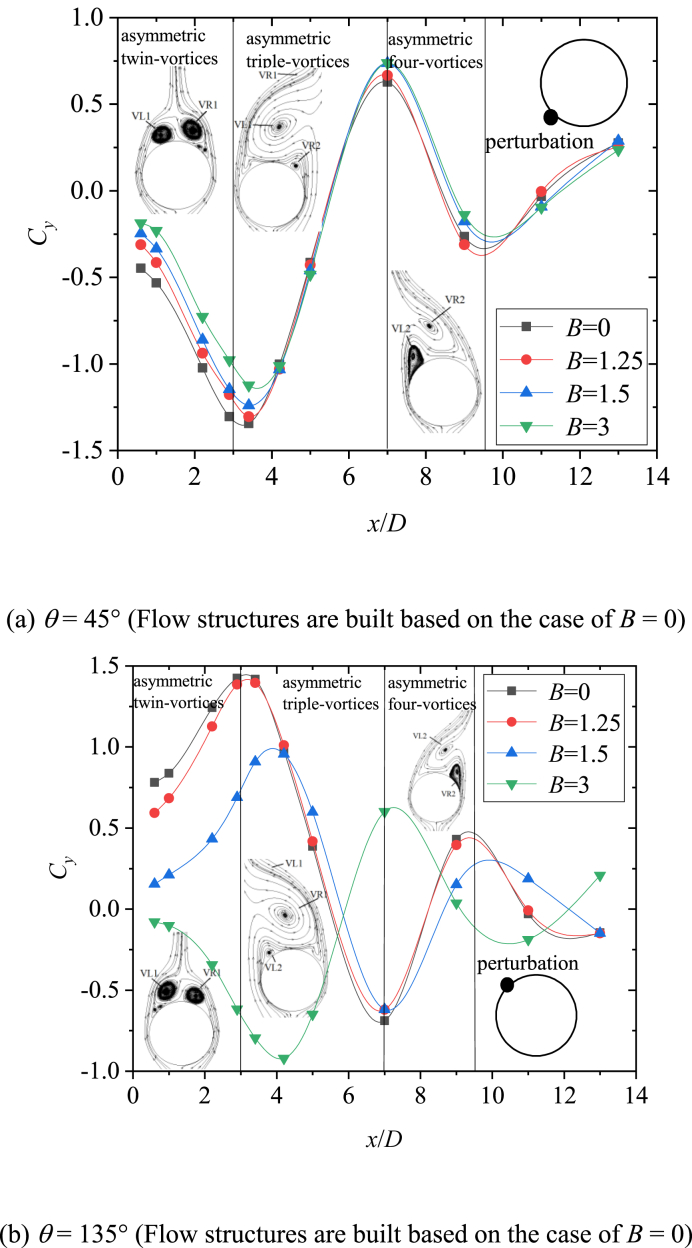
Variation of sectional side-force *C*_*y*_ with *x*/*D* and the corresponding flow structure dia was conducted at the AoA *α* = 50°).

**Fig. 7:**
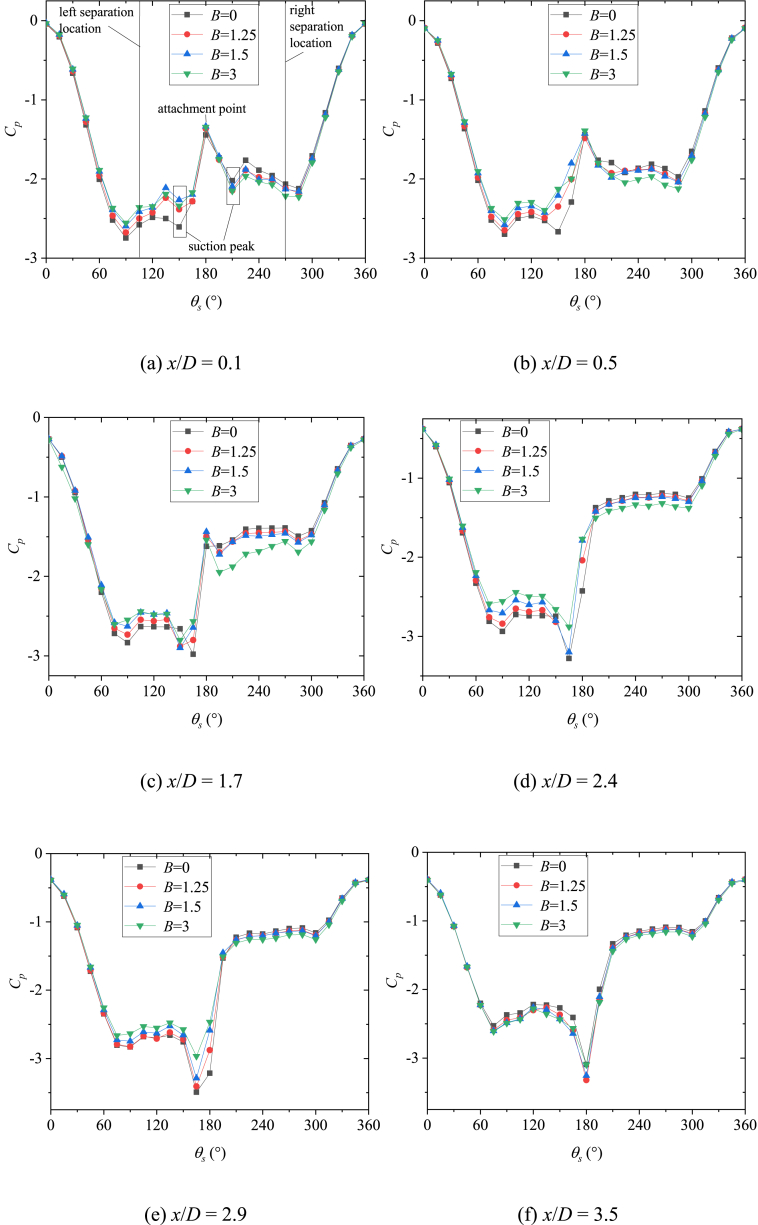
Variations of *C*_*p*_ with sectional angle *θ*_*s*_ at *θ* = 45°.

**Fig. 8:**
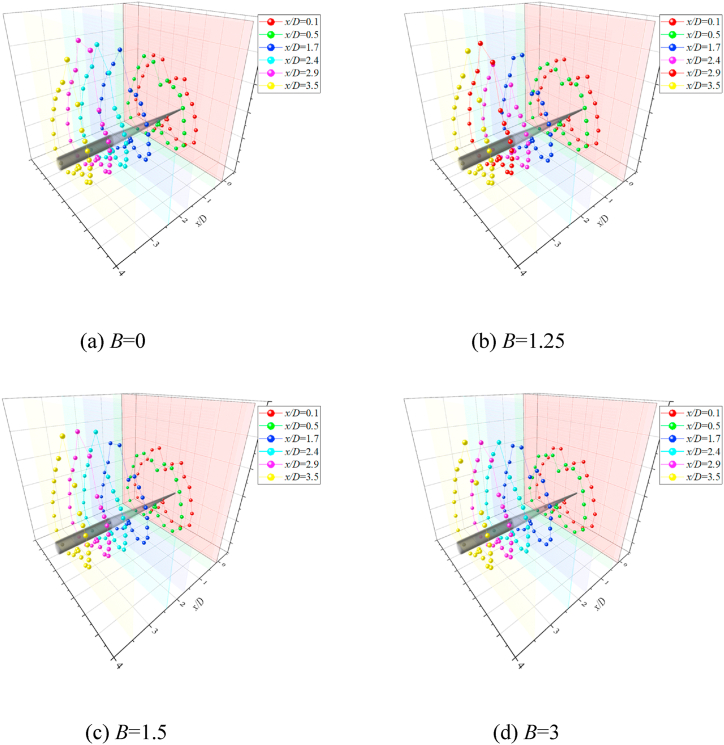
3-D Contour plots of *C*_*P*_ Vs *θ*_*s*_ along *x*/*D* at *θ* = 45°.

**Fig. 9:**
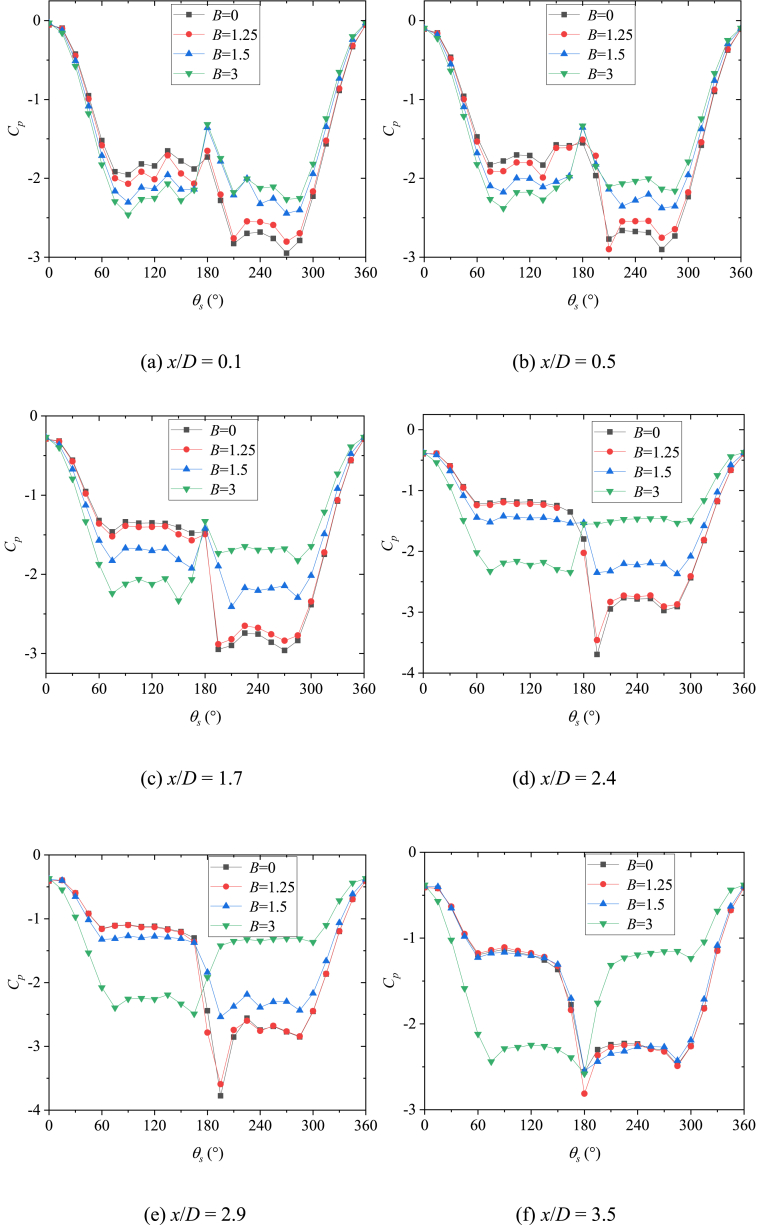
Variations of *C*_*p*_ with sectional angle *θ*_*s*_ at *θ* = 135°.

The behavior of side-forces reflected the pattern of the asymmetric vortices over the slender body [17]. Consequently, the flow was investigated on the basis of the measured sectional side forces in the existence of the artificial perturbation. Fig. 6 presented the variations with *x/D* of the sectional side-force coefficients (*C*_*y*_) at *θ* = 45° and 135°. The two circumferential locations of perturbation produce stable asymmetric flows [37,38], which were beneficial to the study. As shown in Fig. 6(a) for *θ* = 45°, the asymmetric twin-vortices were displayed before the first valley of *C*_*y*_. The artificial perturbation and bluntness jointly affected the asymmetric twin-vortices structure, and then affected the whole asymmetric flow structure. Simultaneously, a close relation between the behavior of the circumferential pressure coefficient distributions and the pattern of asymmetric vortices over the slender body was characterized by Deng [17], and the details of the asymmetric flow can be shown in the pressure distribution. Therefore, Fig. 7 presented the variations of pressure coefficient *C*_*p*_ with sectional angle *θ*_*s*_ at the sections with asymmetric twin-vortices structure. At *x*/*D* = 0.1 (Fig. 7(a)), the pressure distribution curves of four bluntness (*B*) were similar, indicating that the flow structures under the four *B* were similar. The reattachment pressure peak appeared at *θ*_*s*_ = 180°, which was a classic characteristic of twin vortices and implied that the asymmetric twin vortices had formed in this section. The left vortex VL1 rolled from the left side of the model at the left separation location was closed to the surface and the right vortex VR1 rolled from the right side at the right separation location was far away from the surface. So *C*_*p*_ was presented as a larger number in 115° ≤ *θ*_*s*_ ≤ 165° was larger than that in 195° ≤ *θ*_*s*_ ≤ 270°, thus inducing negative side-force coefficients (Fig. 6(a)). For four *B*, suction peaks occurred at *θ*_*s*_ = 150° and *θ*_*s*_ = 210°, respectively, indicating that the circumferential locations of the two vortices (VL1 and VR1) were not affected by *B*. However, with the increase of *B*, *C*_*p*_ increased in 115° ≤ *θ*_*s*_ ≤ 165° and decreased in 195° ≤ *θ*_*s*_ ≤ 270°, indicating that the asymmetry of the two vortices decreased. For *B* = 0, the smooth pressure distribution in 115° ≤ *θ*_*s*_ ≤ 165° indicated that VL1 had been completely separated from the surface. For *B* = 1.25, 1.5 and 3, the obvious peaks occurred at *θ*_*s*_ = 135°, and the kurtosis of the peak increased with the increase of *B*. These observations indicated that nose blunting inhibited the development of vortex and its asymmetry. As VL1 and VR1 developed along the axis, the pressure distributions in 115° ≤ *θ*_*s*_ ≤ 165° showed the smooth structures for four *B*, indicating that VL1 and VR1 have been completely separated from the surface. Then Fig. 7(b)–(f) presented the developments of twin vortices along *x*/*D* until the first valley of *C*_*y*_ in Fig. 6(a). Then VR1 rapidly developed and disengaged from the surface, and a new vortex VR2 complemented its position. Asymmetric triple vortices were occurred between the first valley and the first peak, the side-force initially decreased and then increased in reverse with the development of three vortices (VL1, VR1, and VR2). VL1 then rolled up and VL2 formed, thereby resulting in the flow to evolve into asymmetric four vortices between the first crest and the second valley. Thus, the flow was characterized as a complicated asymmetric multi-vortex structure. The more detailed evolution of asymmetric flow over a slender body along *x*/*D* were depicted in detail by Deng et al. [17] and Qi et al. [39]. Fig. 8 presented the 3-D contour plots of *C*_*P*_ vs *θ*_*s*_ along *x*/*D* at *θ* = 45°, which were the stereoscopic displays of pressure in Fig. 7. The effect of *B* was clearly presented in Figs. 8(a)-9(d).

**Fig. 10:**
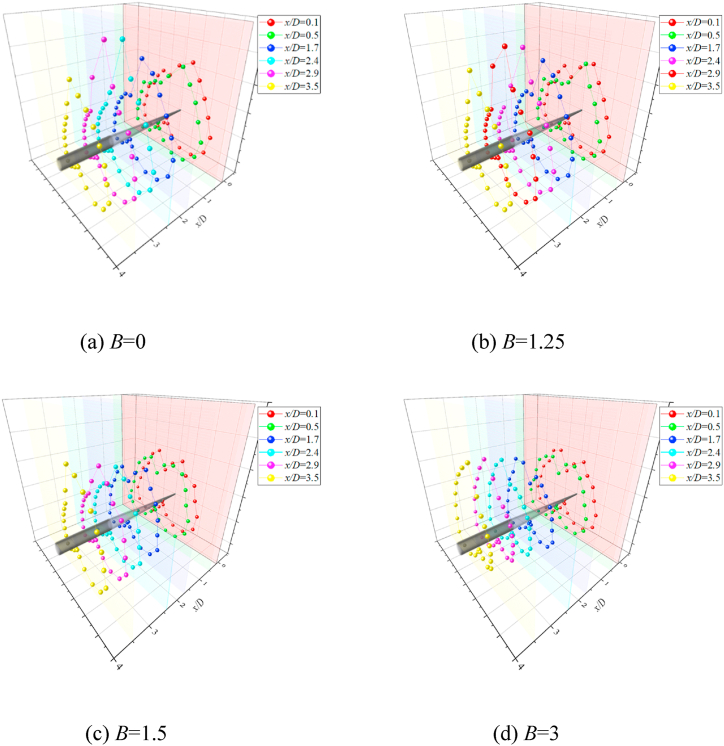
3-D Contour plots of *C*_*P*_ Vs *θ*_*s*_ along *x*/*D* at *θ* = 135°.

When *θ* = 135° as shown in Fig. 6(b), compared to *θ* = 45° in Fig. 6(a), the opposite asymmetric flow pattern was presented, then induced the opposite developments of *C*_*y*_ along the *x*/*D* for the cases of *B* = 0, 1.25 and 1.5. However, similar developments of flow and its corresponding side-force were shown for the cases of *B* = 3. Therefore, the patterns of asymmetric flow and side-force development were related to the circumferential location (*θ*) of the perturbation and bluntness (*B*). Note that the magnitude of *C*_*y*_ at *x*/*D* = 3 decreased as the *B* increases. The observations were consistent with the studies conducted by Keener [23] and Wang [24], that the blunting of the nose can finitely reduce the asymmetry of the flow over a slender body. Fig. 9 presented the variations of pressure coefficient *C*_*p*_ with sectional angle *θ*_*s*_ at the sections with asymmetric twin-vortices structure. For *B* = 0 and 1.25, the *C*_*p*_ showed a pulsating distribution in 90° ≤ *θ*_*s*_ ≤ 180° and the reattachment pressure peak at *θ*_*s*_ = 180° was small at *x/D* = 0.1 (Fig. 9(a)). These observations indicated that the left vortex had not formed completely. Since the perturbation located at *θ* = 135°, where was the left and leeward side of the nose tip, VL formed at *θ*_*s*_ ≈ 90° with a weak vorticity then encountered the perturbation at *θ*_*s*_ = 135°. The perturbation pushed VL away from the surface, so there was no obvious suction peak in 90° ≤ *θ*_*s*_ ≤ 180°. With the evolution of asymmetric flow along *x*/*D*, VL had a fully development at *x*/*D* = 0.5 (Fig. 9(b)) and induced a suction peak at *θ*_*s*_ = 135°. Since VL was far away from the surface, VL was a high vortex compared to VR, so the twin-vortices structure formed was opposite to that with *θ* = 45° (Fig. 6). After the formation of the initial twin-vortices structure, the evolution of asymmetric flow along the model axis was unified into the complex multi-vortices structure built by Deng et al. [17] and Qi et al. [39]. For *B* = 1.5 and 3, the reattachment pressure peak at *θ*_*s*_ = 180° indicated that the twin-vortices structure had been formed at *x*/*D* equaling 0.1 (Fig. 9(a)). According to the discussion about the formation of asymmetric flow over a blunt-nosed slender body by Qi et al. [37], it can be inferred that the perturbation moved from downstream (as *B* = 1.5) to upstream (as *B* = 3) of the starting point of the separation. As shown in Fig. 9(a)–(f), *C*_*p*_ in 90° ≤ *θ*_*s*_ ≤ 150° had a smaller magnitude than that in 210° ≤ *θ*_*s*_ ≤ 270° for *B* = 1.5, however the magnitude was reversed for *B* = 3. These observations implied that compared with VR, VL changed from high vortex (*B* = 1.5) to low vortex (*B* = 3). Fig. 10 presented the 3-D contour plots of *C*_*P*_ vs *θ*_*s*_ along *x*/*D* at *θ* = 135°, which were the stereoscopic displays of pressure in Fig. 9. The effect of *B* was clearly presented in Fig. 10(a)–(d).

**Fig. 11:**
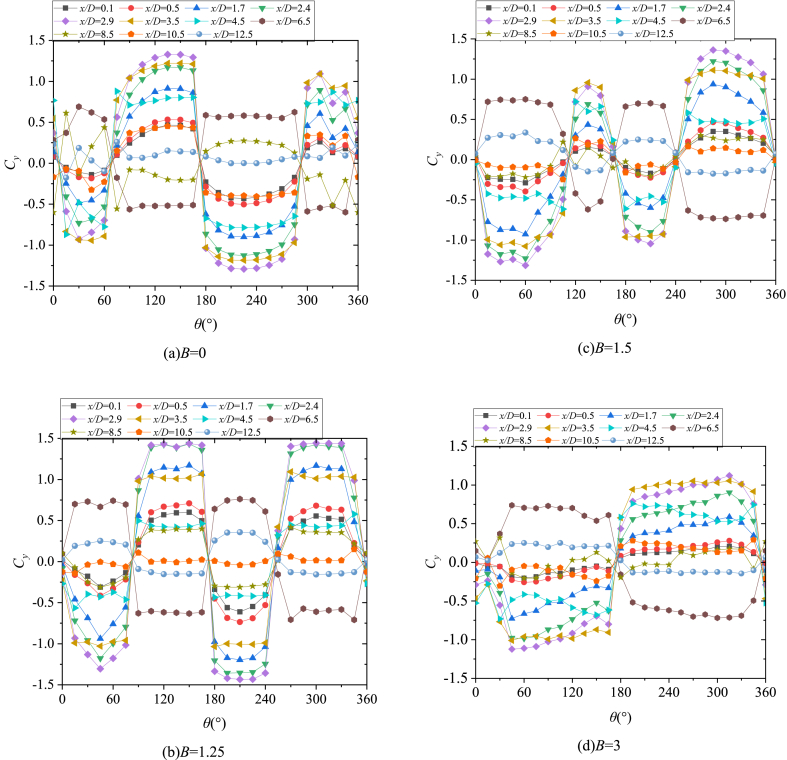
Variations with *θ* of the sectional side-force coefficients *C*_*y*_ in the presence of an artificial perturbation.

**Fig. 12:**
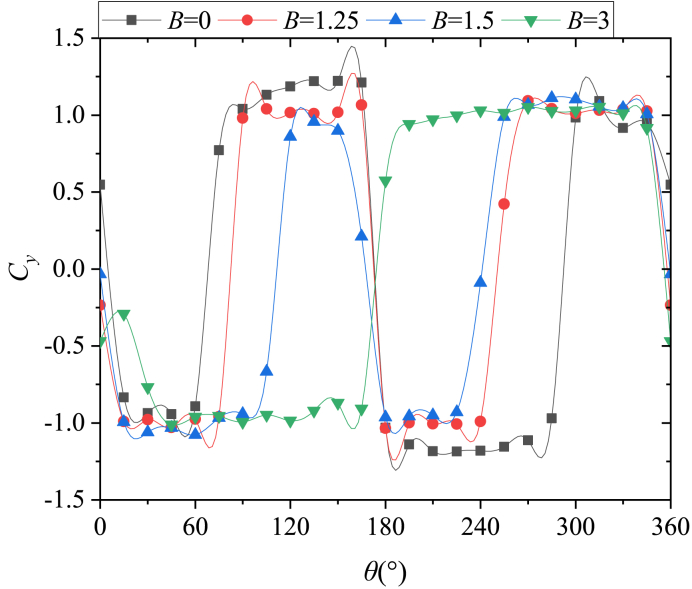
Variations with *θ* of *C*_*y*_ in the presence of artificial perturbation at *x/D* = 3.5.

**Fig. 13:**
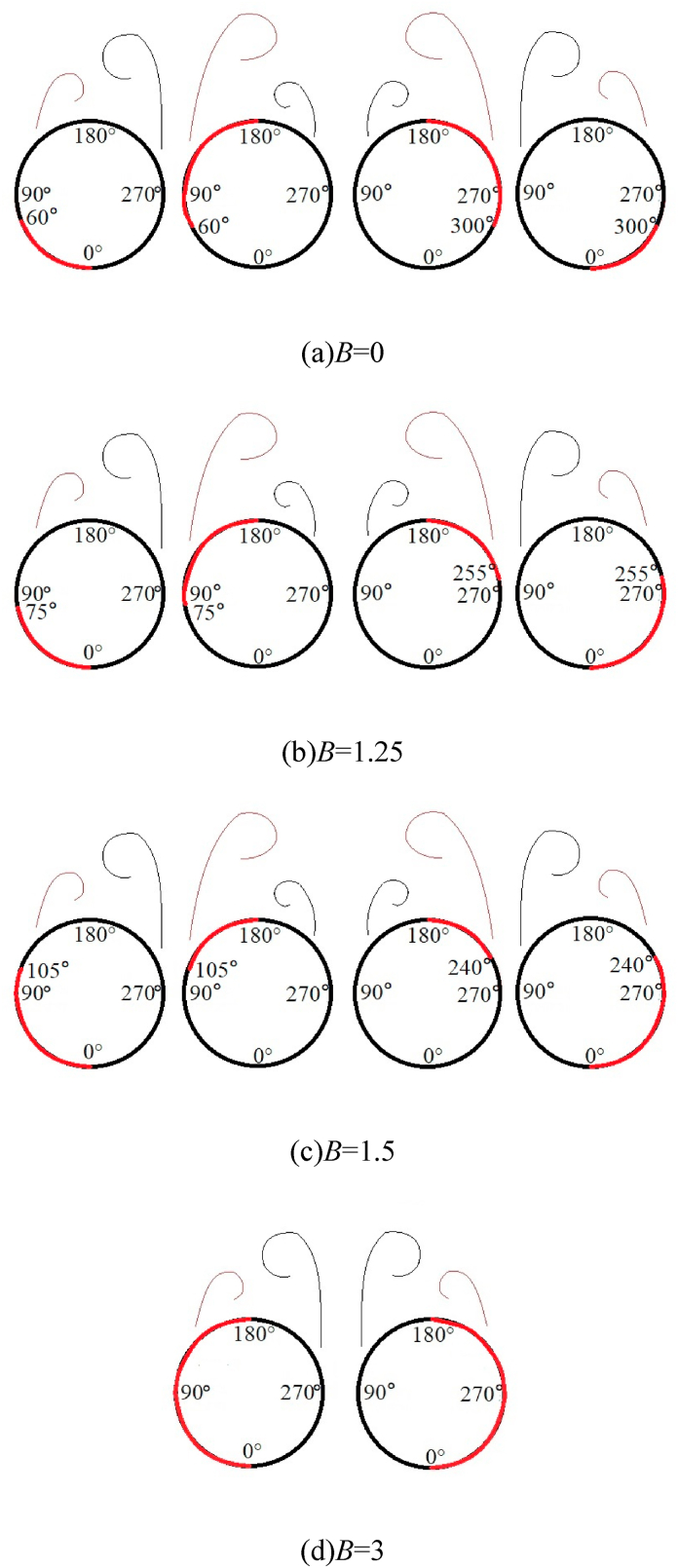
Patterns of asymmetric vortices at different circumferential locations of perturbation for different bluntness *B*; the red line indicates the circumferential interval of the perturbation (*x/D* = 3.5). (For interpretation of the references to colour in this figure legend, the reader is referred to the Web version of this article.)

Based on the discussion in Fig. 6, Fig. 7, Fig. 8, Fig. 9, Fig. 10, *B* and *θ* were two important parameters affecting the asymmetric flow over the slender body. Fig. 11 presented the variations with *θ* of *C*_*y*_ at all the testable *x*/*D* for different *B*. The double-periodic square-wave shapes were presented when *θ* was increased from 0° to 360° for *B* = 0, 1.25, and 1.5 (Fig. 11 [a], [b], and [c]). However, the single-periodic square-wave shapes were presented for *B* = 3 (Fig. 11 [d]). When the perturbation was placed at the symmetrical locations about the *xz*-plane, the side-force had a uniform magnitude but was opposite in directions. For instance, considering that *x*/*D* = 3.5, as shown in Fig. 12, the switches from the negative *C*_*y*_ to the positive *C*_*y*_ occurred at *θ* ≈ 60° and 300° when *B* = 0. *C*_*y*_ was negative and the side-force was in the negative *y*-direction when the perturbation was located at 0° < *θ* < 60° and 180° < *θ* < 300°. Meanwhile, *C*_*y*_ was positive and the side-force was along the positive *y*-direction when the perturbation was located at 60° < *θ* < 180° and 300° < *θ* < 360°. With the increase of *B*, the switches kept moving toward *θ* = 180° (*θ* ≈ 75° and 255° for *B* = 1.25; *θ* ≈ 105° and 240° for *B* = 1.5) until they overlapped with *θ* = 180° at *B* = 3. Then the double-periodic square-wave shape for *θ* increasing from 0° to 360° turned into a single-periodic square-wave shape with the increased *B*. Likewise, the magnitude of *C*_*y*_ decreased with the increase of *B*, which confirmed the discussion in Fig. 6. Fig. 13 presented the patterns of asymmetric twin-vortices at different circumferential perturbation locations for different *B* (Fig. 13(a)-[d]) based on the relationship between asymmetric flow pattern and sectional side-force discussed by Deng et al. [17]. The circumferential effect of perturbation on asymmetric flow turned from double-to single-periodic.

### 3.2 Effects of bluntness on the tip separation

**Fig. 14:**
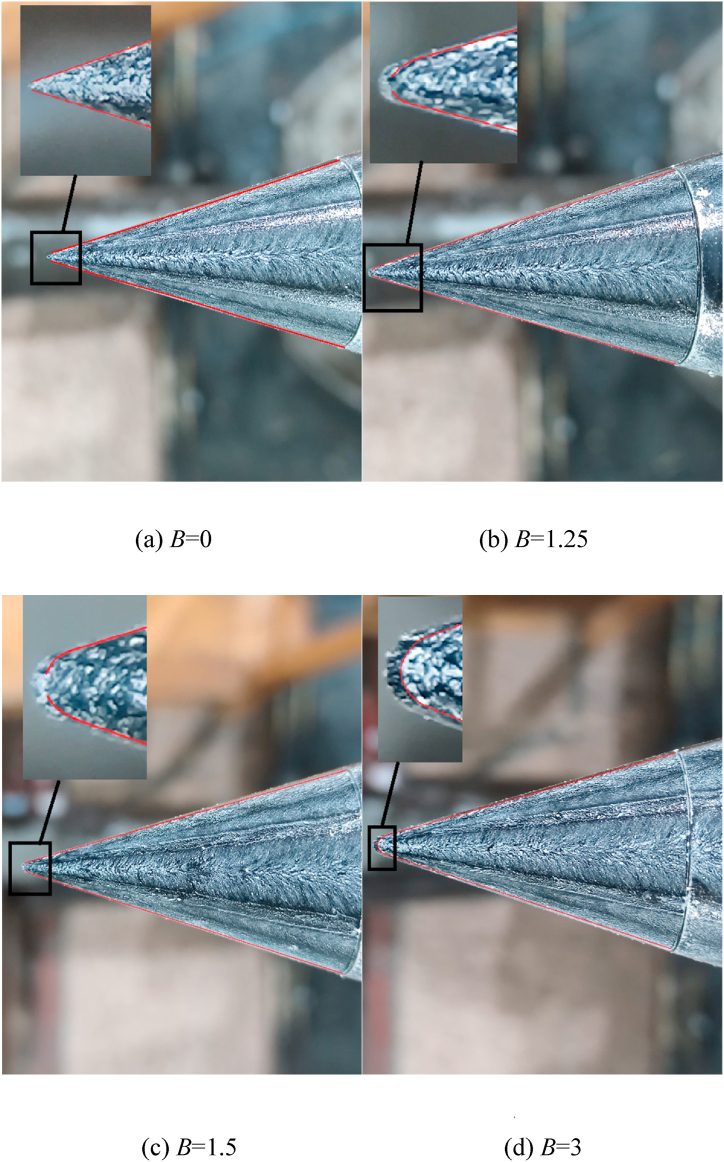
Oil surface visualization at the tip of the nose.

The perturbation near the tip of the nose mainly controlled the pattern of the asymmetric twin-vortices (see Fig. 6) by initially affecting the separation line structure on the tip, and finally affecting the whole asymmetric flow [37,38]. The separation lines were the vanishing locations of the wall shear stress. Fig. 14 presented the oil surface visualization at the nose tip in the absence of a perturbation based on the oil surface visualization technology used by Lopera [40] in analyzing the asymmetric flow over a slender body, and the separation lines were highlighted in red. Based on the characteristics of the open- and close-type separation discussed by Wang [24], for *B* = 0, 1.25, and 1.5, the left and right separation lines were separated at the nose tip. The open-type separation was presented in Fig. 14(a), [b], and [c]). For *B* = 3, the left and the right separation lines were closed at the tip of the nose, and the separation types of the horseshoe were presented in Fig. 14(d)). The characteristics of the separation were named as close-type separation.

**Fig. 15:**
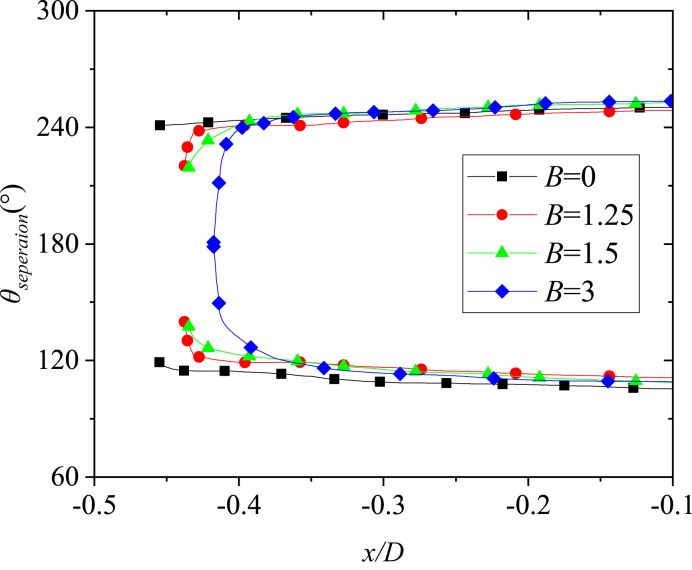
Variations with *x*/*D* of the separation locations at the tip of the nose.

Fig. 15 presented the circumferential locations of separation lines on the nose tip. The starting positions of the separation lines on either side of the model kept approaching the circumferential separation position *θ*_*separation*_ = 180° with the increase of *B*. When *B* = 3, the two starting points of separation lines on both sides were closed, thereby forming a close-type separation with the characteristics of horseshoe-type. The starting points of the separation lines on both sides of the tip were closely related to the switching circumferential angles of positive and negative side-forces as implied in these observations. As the two starting points get closer to *θ*_*separation*_ = 180° with the increase of *B* (Fig. 14, Fig. 15), the switches on both sides get closer to *θ* = 180° (Fig. 11, Fig. 12). When the two starting points overlapped at *θ*_*separation*_ = 180°, thereby forming a close-type separation at *B* = 3, the two switched overlap at *θ* = 180°, the double period become single period. Starting points moving with the changing *B* was because of the crossflow that dominated the separation over the tip when *B* = 0. When *B* ＞ 0，the axial flow generated over the blunt-nose, the starting points were affected by a combination of cross- and axial flows. With the increase of *B*, the axial flow over the nose increased, and thus the starting points of separation lines turned to *θ*_*separation*_ = 180° until it forms a close-type separation.

### 3.3 Analysis on periodic characteristics of perturbed flow

**Fig. 16:**
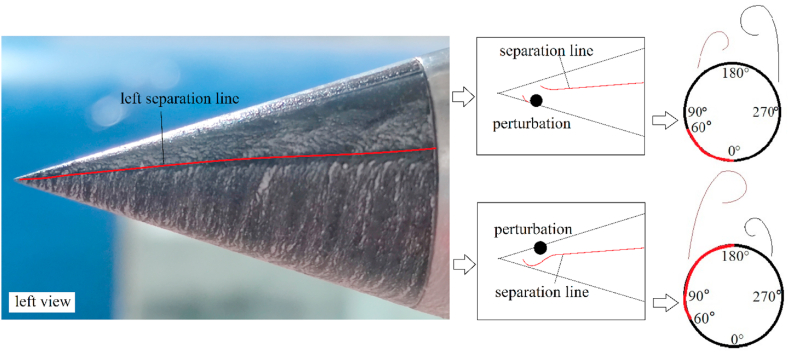
The schematic diagram of double-periodic mechanism of perturbed flow over a pointed-nosed slender body with *B* = 0.

For *B* = 0, the perturbation was located at the axial location *L*_*p*_ = 0.015 *D*, thus, the perturbation located at *x*/*D* = −0.445 in Fig. 15. According to Qi et al. [38], the perturbation affected the shear layer separation at the nose tip, that was, affecting the ipsilateral separation line structure and subsequently managing the pattern of the asymmetric flow. Fig. 16 presented the double-periodic mechanism of perturbed flow over a pointed-nosed slender body with *B* = 0. When the perturbation was located at the circumferential location 0° ≤ *θ* ≤ 60°, locating on the windward side of the separation line. The perturbation blocked the development of shear layer separation near the tip, and the separation line was divided into two parts by the perturbation. The broken separation line moved to the leeward side, thereby leading to the axial position, where the vortex rolled, and moved backward along the axis, thereby resulting in the formation of a low vortex as shown in Fig. 16. Therefore, the asymmetric twin-vortices structure was formed, the low vortex was on the same side as the perturbation, and the corresponding side-forces were negative. When the perturbation was located at the circumferential of locations 60° ≤ *θ* ≤ 180°, locating on the leeward side of the separation line. The perturbation pushed the separation line to the windward side, thereby promoting the boundary layer separation and the location where vortex rolls from the surface moves forward as shown in Fig. 16. Initially, the vortex was lifted from the wall surface and fully developed, becoming the high vortex. Hence, for the asymmetric twin-vortices structure formed, the high vortex was on the same side with the perturbation when 60° ≤ *θ* ≤ 180°. When the perturbation was located at the right side of the model, the asymmetric flow pattern induced by the perturbation was symmetric with that produced by the symmetrical perturbation at the left side. Thus, the asymmetric flow and its corresponding side-force coefficient changed in a double period when the roll angle increased from 0° to 360°.

For *B* = 1.25 and 1.5, the perturbation was located at the axial location *γ* = 10°, hence, the perturbation was located at *x*/*D* = −0.446 and −0.443 in Fig. 15. Open-type separation was still the structural characteristic of the separation line. The influence of perturbation on asymmetric flow and the double periodic generation mechanism were the same as presented in Fig. 16. However, with the increase of *B*, the axial flow over the tip of the nose increased, hence, the separation lines developed towards *θ*_*separation*_ = 180°, thereby resulting in the decrease of the leeward area and increase of the windward area of the separation lines. Therefore, the relationship between the circumferential perturbation location and the asymmetric flow pattern, together with the corresponding side-force presenting the changes with *B* were shown in Fig. 11, Fig. 12, Fig. 13.

**Fig. 17:**
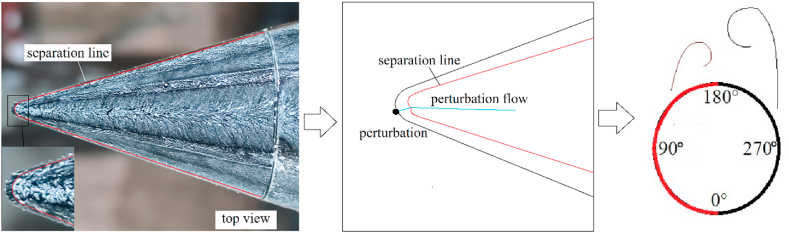
Schematic diagram of the single-periodic mechanism of the perturbed flow over a blunt-nosed slender body with *B* = 3.

For *B* = 3, the perturbation was located at the axial location *γ* = 10°, hence, the perturbation located at *x*/*D* = −0.426 in Fig. 15. Close-type separation was the structural characteristic of separation line and the perturbation was located upstream of the separation line. According to Qi et al. [37], the perturbation flow detaching from the perturbation surface split the shear layer detached from the nose into two asymmetric portions, then managed the asymmetric flow pattern. The single-periodic mechanism of perturbed flow over a blunt-nosed slender body with *B* = 3 was presented in Fig. 17. The close-type separation was presented by the oil surface visualization in the photograph. When a perturbation was added onto the tip of the nose, as shown in the schematic diagram, the flow separated from the perturbation surface and rolled up as perturbation flow. The perturbation flow divided a separation of the shear layer (shown as a separation line) into two asymmetric parts (long and short). When the perturbation was located at the left side of the nose, the split location of the separation line was on the left, thus, the short and the long part of the separation were on the left and right, respectively. The long part on the opposite side to the perturbation rolled up a vortex sooner than that short part on the same side to the perturbation. The differences in the two parts resulted in an asymmetric vortices pattern. Similarly, when the perturbation was located at the right side of the model, the pattern of asymmetric flow produced by the perturbation was symmetric with that produced by the symmetrical perturbation at the left side. Thus, the asymmetric flow and its corresponding side-force changed in a single period when the roll angle increased from 0° to 360°.

**Fig. 18:**
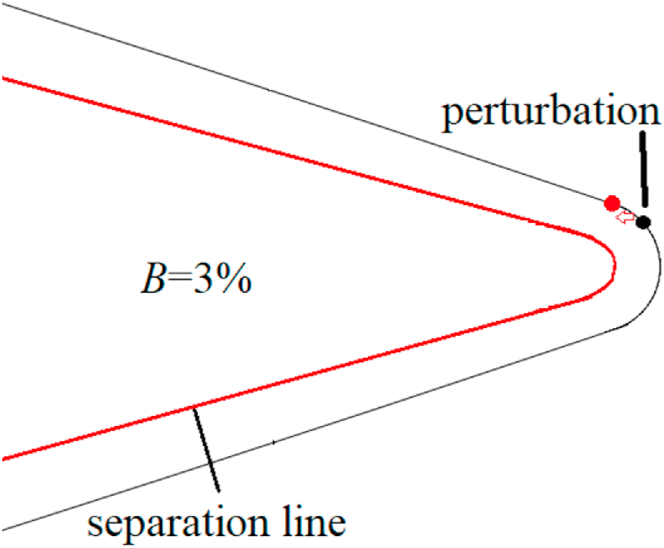
Schematic diagram of changing perturbation location.

**Fig. 19:**
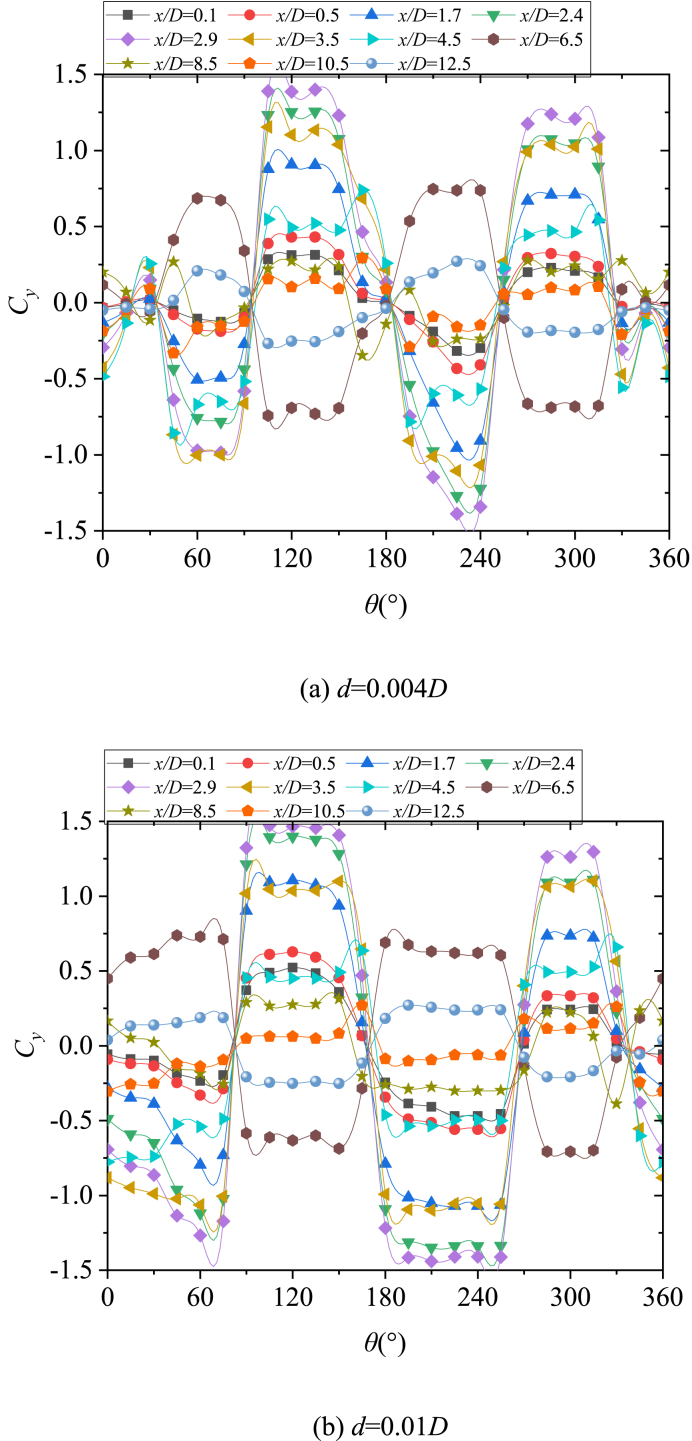
Variations with *θ* of the sectional side-force coefficients *C*_*y*_ in the presence of an artificial perturbation for *B* = 3.

Based on the presentation of Fig. 16, Fig. 17 for the open-type separation, the perturbation was involved in shear layer separation, wherein it directly affected the formation of separation lines and thus managed the asymmetric flow pattern. However, for close-type separation, the perturbation that was located upstream of the separation line affected the separation line through the micro-flow separated from the surface of the perturbation, thus managing the asymmetric flow pattern. To prove this, for *B* = 3, the perturbation moved to *x*/*D* = −0.4 (*γ* = 75°) from *x*/*D* = −0.426 (*γ* = 10°) (refer to Fig. 15). That was, the perturbation moved from the upstream of separation line to the axial location where the separation line just generated, as shown in Fig. 18. In addition, the perturbation directly affected the shear layer separation on either side of the nose, similar to the open-type separation (Fig. 18). In addition, the perturbation directly affected the shear layer separation on either side of the nose, similar to the open-type separation (Fig. 18). Fig. 19 presented the variations with *θ* of the sectional side-force coefficients *C*_*y*_ in the presence of a perturbation. Fig. 19(a) showed the case of perturbation with the diameter of 0.004*D*, which was similar with that used in Fig. 11. The relationship between side-force and a circumferential location of perturbation changed from a single period to an irregular double period. The sensitivity of asymmetric flow to perturbation weakened as the perturbation moved backward [16], thus, the double period presented irregular fluctuation. Meanwhile, Fig. 19(b) showed the case of perturbation with a diameter of 0.01*D*, which had a greater ability to manage asymmetric flow patterns. A double period which was similar with that for the open-type separation was presented.

## 4 Conclusions

The pressure measurement experiment and surface oil-flow visualization techniques were carried out to investigate the periodic characteristics of perturbed flow over a slender body. The suppression of flow asymmetry by nose blunting was clarified by analyzing the change of the starting point of the separation with increasing bluntness (*B*). Meanwhile, the mechanism of switching from open-to close-type of separation with increasing *B* were studied and explained in detail. The critical *B* of separation pattern switching from open-type to close-type located between 1.5 and 3. Finally, by analyzing the influence of perturbation on the open-type separation line and the close-type separation line, the double and single periodic characteristics of perturbed flow over pointed- and blunt-nosed slender body were separately revealed. Results showed that the axial flow increased with the increase of bluntness, resulting in the open-type separation turning into a close-type separation. For the open-type separation, the perturbation was located behind the starting point of the separation line and directly participated in the shear layer separation. In addition, it induced the double period between the circumferential location of perturbation and the perturbed flow. Meanwhile, for the close-type separation, the perturbation was located upstream of the separation line and affected the separation line through the micro-flow separated from its surface. Moreover, it induced a single period between the circumferential location of perturbation and the perturbed flow.

## Author contribution statement

Li Zhao: Performed the experiments; Wrote the paper.

Yankui Wang: Contributed reagents, materials, analysis tools or data.

Zhongyang Qi: Conceived and designed the experiments; Analyzed and interpreted the data.

## Data availability statement

Data will be made available on request.

## Declaration of competing interest

The authors declare that they have no known competing financial interests or personal relationships that could have appeared to influence the work reported in this paper.

## References

[1] Thomson K.D., Morrison D.F. (1971). The spacing, position and strength of vortices in the wake of slender cylindrical bodies at large incidence. J. Fluid Mech..

[2] Hunt B. (1982). 9th Atmospheric Flight Mechanics Conference.

[3] Bridges D.H., Hornung H.G. (1994). Elliptic tip effects on the vortex wake of an axisymmetric body at incidence. AIAA J..

[4] Hsieh T., Wang K.C. (1996). Three-dimensional separated flow structure over a cylinder with a hemispherical cap. J. Fluid Mech..

[5] Xu Y., Chen S., Zhou H. (2021). Analysis of the Magnus moment aerodynamic characteristics of rotating missiles at high altitudes. Int. J. Aer. Eng..

[6] Tasci M.O., Pektas M.C., Tumse S., Karasu I., Sahin B., Akilli H. (2021). The impact of the pitching motion on the structure of the vortical flow over a slender delta wing under sideslip angle. J. Visual.

[7] Karasu I., Tumse S., Tasci M.O., Sahin B., Akilli H. (2021). Near-surface particle image velocimetry measurements over a yawed slender delta wing. Proc. Inst. Mech. Eng. G J. Aerosp. Eng..

[8] Oguz Tasci M., Tumse S., Sahin B. (2022). Vortical flow characteristics of a slender delta wing in ground effect. Ocean Eng..

[9] Pick G. (1971).

[10] Levy Y., Hesselink L., Degani D. (1995).

[11] Ericsson L., Reding J. (1980).

[12] Ericsson L., Reding J. (1985).

[13] Calarese W. (1981).

[14] Wei K., Chen S., Xu Y., Tang D. (2022). Influence of roughness on the asymmetric flow field of a slender body. Int. J. Aer. Eng..

[15] Rao D.M. (1979). Side-force alleviation on slender, pointed forebodies at high angles of attack. J. Aircraft.

[16] Chen X.R., Deng X.Y., Wang Y., Liu P.Q., Gu Z.F. (2002). Influence of nose perturbations on behaviors of asymmetric vortices over slender body. Acta Mech. Sin..

[17] Deng X.Y., Liu P.Q. (2000). Study on the characteristics and formation mechanism of asymmetric vortices over a slender body. Acta Mech. Sin..

[18] Deng X.Y., Wang Y.K. (2004). Asymmetric vortices flow over slender body and its active control at high angle of attack. Acta Mech. Sin..

[19] Deng X.Y., Tian W., Ma B.F., Wang Y.K. (2008). Recent progress on the study of asymmetric vortex flow over slender bodies. Acta Mech. Sin..

[20] Meng X.S., Long Y.X., Wang J.L., Liu F., Luo S.J. (2018). Dynamics and control of the vortex flow behind a slender conical forebody by a pair of plasma actuators. Phys. Fluids.

[21] Wang Q.T., Cheng K.M., Gu Y.S., Li Z.Q. (2018). Continuous control of asymmetric forebody vortices in a bi-stable state. Phys. Fluids.

[22] Chapman G.T., Keener E.R., Malcolm G. (1976).

[23] Keener E.R., Chapman G.T., Cohen L. (1977).

[24] Wang G., Deng X.Y., Liu P.Q. (2004). Influence of nose bluntness on behaviors of multi-asymmetric vortices flow over slender body. Acta Mech. Sin..

[25] Kamakoli G.H., Mansour K. (2021). Determining the side force appearance and its magnitude over a slender body at high angle of attack. Thermophys. Aeromechanics.

[26] Matthew Z., Demetri T. (1997).

[27] Bernhardt J.E., Williams D.R. (2000). Closed-loop control of forebody flow asymmetry. J. Aircraft.

[28] Darden L.A., Komerath N.M. (1995).

[29] Patel M.P., Tilmann C.P., Ng T.T. (2004). Closed-loop missile yaw control via manipulation of forebody flow asymmetrics. J. Spacecraft Rockets.

[30] F.C. Wong, Missile Flight Control Using Micro-actuated Flow Effectors - Review of Fiscal Year 2005/2006 Progress, DRDC Valcartier TN2005-282.

[31] Kumar R., Viswanath P.R., Ramesh O.N. (2005). Nose bluntness for side-force control on circular cones at high incidence. J. Aircraft.

[32] Qi Z.Y., Wang Y.K., Bai H.L., Sha Y.X., Li Q. (2018). Effects of micro-perturbations on the asymmetric vortices over a blunt-nose slender body at a high angle of attack. Eur. J. Mech. B Fluid.

[33] Deng X.Y., Wang G., Chen X.R. (2003). A physical model of asymmetric vortices flow structure in regular state over slender body at high angle of attack. Sci. China, Ser. A.

[34] Leeuw W.C.D., Post F.H., Walter B. (1995). Visual simulation of experimental oil-flow visualization by spot noise images from Numerical Flow Simulation. Visual. Scient. Comp..

[35] Ramakrishna M., Syedhaleem M., Divya C., Gurunathan A. (2018). Flow characteristics over a missile at higher angle of attacks using experiments and simulation. Int. J. Veh. Struct. Syst..

[36] Merzkirch W. (1987).

[37] Qi Z.Y., Zong S.Y., Wang Y.K., Li Q., Wang J.J. (2019). Sources of asymmetric flow over the blunt-nose slender body. Eur. J. Mech. B Fluid.

[38] Qi Z.Y., Zong S.Y., Wang Y.K. (2021). Bi-stable asymmetry on a pointed-nosed slender body at a high angle of attack. J. Appl. Phys..

[39] Qi Z.Y., Wang Y.K., Wang L., Li Q. (2017). Investigation on asymmetric flow over a blunt-nose slender body at high angle of attack. Fluid Dynam. Res..

[40] Lopera J., Ng T.T., Patel M.P., Stucke R. (2007). Forebody geometry effects on the flow of a blunt-nose projectile at high alpha. J. Aircraft.

